# Evaluation of TorsinA as a Target for Parkinson Disease Therapy in Mouse Models

**DOI:** 10.1371/journal.pone.0050063

**Published:** 2012-11-21

**Authors:** Xinru Li, Jenny Lee, Dee Parsons, Karen Janaurajs, David G. Standaert

**Affiliations:** Center for Neurodegeneration and Experimental Therapeutics, Department of Neurology, The University of Alabama at Birmingham, Birmingham, Alabama, United States of America; Hertie Institute for Clinical Brain Research and German Center for Neurodegenerative Diseases, Germany

## Abstract

Parkinson disease (PD) is a common and disabling disorder. No current therapy can slow or reverse disease progression. An important aspect of research in this field is target validation, a systematic approach to evaluating the likelihood that modification of a certain molecule, mechanism or biological pathway may be useful for the development of pharmacological or molecular treatments for the disease. TorsinA, a member of the AAA+ family of chaperone proteins, has been proposed as a potential target of neuroprotective therapy. TorsinA is found in Lewy bodies in human PD, and can suppress toxicity in cellular and invertebrate models of PD. Here, we evaluated the neuroprotective properties of torsinA in mouse models of PD based on intoxication with 1-methyl-4-phenyl-1,2,3,6-tetrahydropyridine (MPTP) as well as recombinant adeno associated virus (rAAV) induced overexpression of alpha-synuclein (α-syn). Using either transgenic mice with overexpression of human torsinA (hWT mice) or mice in which torsinA expression was induced using an rAAV vector, we found no evidence for protection against acute MPTP intoxication. Similarly, genetic deletion of the endogenous mouse gene for torsinA (*Dyt1*) using an rAAV delivered Cre recombinase did not enhance the vulnerability of dopaminergic neurons to MPTP. Overexpression of α-syn using rAAV in the mouse substantia nigra lead to a loss of TH positive neurons six months after administration, and no difference in the degree of loss was observed between transgenic animals expressing forms of torsinA and wild type controls. Collectively, we did not observe evidence for a protective effect of torsinA in the mouse models we examined. Each of these models has limitations, and there is no single model with established predictive value with respect to the human disease. Nevertheless, these data do seem to support the view that torsinA is unlikely to be successfully translated as a target of therapy for human PD.

## Introduction

Parkinson disease (PD) is a progressive neurodegenerative disease which causes a movement disorder characterized by bradykinesia, resting tremor, rigidity, and postural instability along with non-motor features which include autonomic dysfunction and cognitive impairment. There is at present no treatment with established efficacy in preventing or slowing the progression of PD, and development of such treatments is a high priority for the field. A variety of potential approaches to such “neuroprotective” treatments have been described, but most have not been carefully evaluated in either preclinical models of PD or in human patients [1]. Progress towards neuroprotection will require development of improved approaches to “target validation”: development of a systematic approach to evaluate the likelihood that modification of a certain molecule, mechanism or biological pathway may be useful for the development of pharmacological or molecular treatments for the disease [2].

The protein torsinA has been proposed as a potential target of PD therapy, based on evidence from cellular systems, animal models and human postmortem studies. TorsinA was first identified as the cause of a human genetic disorder, DYT1 dystonia [3]. Although there is evidence suggesting that dystonia, like the motor symptoms of PD, arises from basal ganglia dysfunction, in human DYT1 dystonia there is no clear evidence for neurodegeneration or neuron loss, and the symptoms are believed to arise as a result of abnormal plasticity and defects in microcircuitry of the neuronal systems [4], [5]. The dystonia-causing mutation is a 3-bp deletion in the TOR1A gene, that deletes a glutamic acid residue in the C-terminal coding region of the protein torsinA. The protein is a member of the AAA+ (ATPases Associated with a variety of cellular Activities) superfamily. Members of this protein family typically form multimeric assemblies, and participate in protein folding and chaperone processes [4], [6]. On a cellular level, torsinA is a resident protein in the endoplasmic reticulum (ER) and nuclear envelope (NE), and seems to be involved in regulating the interactions of the NE and ER compartments with the cytoskeleton [7]–[10]. One of the proteins modulated by torsinA, both *in vitro* and in invertebrate models, is the dopamine transporter (DAT), which is sequestered intracellularly by high levels of torsinA expression [11], [12].

Evidence linking torsinA to PD has been generated by a number of different laboratories. *In situ* hybridization studies of torsinA mRNA in human brain demonstrate high-level expression of the transcript in dopamine neurons [13]. TorsinA appears to be able to interact with alpha-synuclein (α-syn), a protein with a central role in the pathophysiology of PD. Alpha-synuclein is the primary constituent of Lewy bodies, intraneuronal inclusions which are invariably present in dopamine neurons in human PD, and torsinA is also present within these inclusions. Moreover, experiments using fluorescence resonance transfer have shown that within Lewy bodies torsinA and α-syn are closely associated [14]. In an H4 neuroglioma cell model, torsinA is a potent suppressor of α-syn aggregation and toxicity [15]. In a *Caenorhabditis elegans* model, overexpression of torsinA in neurons results in dramatic suppression of neurodegeneration caused by overexpression of α-syn, and protection against the dopaminergic neurotoxin 6-hydroxydopamine (6-OHDA) [12]. It has been proposed that these protective effects may arise from the chaperone-like properties of torsinA, which may enable it to act on misfolded proteins to cause either refolding or degradation.

While these data from cellular, invertebrate and human postmortem studies are encouraging, a critical step is evaluation of potential targets in intact mammalian systems. The goal of this study is to address this gap in knowledge and to evaluate torsinA as a potential neuroprotective agent in mouse models of PD. There is at present no single animal model which recapitulates all of the etiological and pathophysiological features of human PD. We have selected two distinct mouse models, based on different mechanisms, for this validation study: acute 1-methyl-4-phenyl-1,2,3,6-tetrahydropyridine (MPTP) intoxication [16], and chronic α-syn overexpression induced by a recombinant adeno-associated viral (rAAV) vector (rAAV-SYN) [17]. We have employed several techniques to manipulate the expression of torsinA in this system. Using an existing Dyt1-loxP (“floxed”) homozygote mouse (loxP) [18] and rAAV-mediated delivery of Cre recombinase (Cre) [19], we evaluated whether knockout of torsinA enhances sensitivity to MPTP in mice. Furthermore, we used an existing mouse line [20], a transgenic overexpressing wild type human torsinA (hWT), to determine whether overexpression of wild type torsinA is neuroprotective in the MPTP or rAAV-SYN mouse PD model. The endpoints of each of these studies are based on direct determination of the number of tyrosine-hydroxylase (TH) positive neurons remaining, as well as neurochemical assessment of the striatal content of dopamine (DA) and its metabolites.

## Results

### Effects of TorsinA Overexpression Using an rAAV Vectors in an Acute MPTP Intoxication Model

To determine whether torsinA confers protection against MPTP, high-titer rAAV8 vector containing the human wild type torsinA gene was stereotaxically injected unilaterally into the SN in male adult WT mice. Control mice received an identical injection of rAAV8 vector expressing GFP. One month after virus injection, the mice were treated with MPTP, using 4 doses of the toxin administered in a single day (see Methods). Mice were euthanized at 14 days post-MPTP injection. To maximize the value of these experiments, we divided the forebrain from the midbrain in the fresh state. The striata were dissected and frozen separately, while the entire midbrain was fixed by immersion in paraformaldehye and later frozen and sectioned on a sliding microtome for stereology study. This study also included a group of mice which did not receive any viral vector injection and were treated only with saline vehicle, to assess the efficacy of the MPTP lesion.

**Figure 1:**
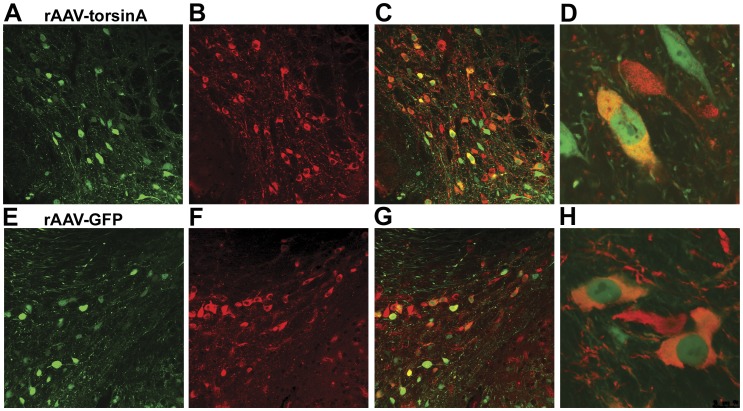
Human torsinA overexpression in the substantia nigra (SN) of wild-type mice after injection of the rAAV8-torsinA or rAAV8-GFP vectors. The images were obtained from the MPTP treated animals. SN sections were double stained for TH (red) and either torsinA or GFP (green). Top panels, TorsinA expression (A) in TH positive neurons of the SN (B) at 6 weeks following intracranial injection of rAAV8-TorsinA. Merged images (C and D) indicate extent of TH cells transfected with torsinA. Bottom panels, similar images demonstrating rAAV8-induced expression of GFP (E) in TH positive nigral neurons (F); G and H are merged images demonstrating colocalization.

Viral gene expression was examined immunohistochemically using an antibody that specifically recognizes human normal torsinA or an anti-GFP antibody in sections from the MPTP treated animals from these experiments. In the rAAV8-torsinA injected animals, this staining revealed prominent human torsinA protein expression within TH positive neurons in the SN on the side of the injection (**Figure 1A**) and no significant staining on the contralateral side (not illustrated). A similar pattern of expression was detected in the rAAV8-GFP injected mice, with prominent GFP expression in TH nigral neurons (**Figure 1E**). Double immunostaining with TH and torsinA or TH and GFP antibodies revealed that in most animals at least half of TH positive neurons in a given section of the SN clearly expressed the rAAV encoded protein. The torsinA immunoreactivity was most prominent in the cytoplasm but could also be detected in the nucleus of neurons; this differs from the native endoplasmic reticulum location of the protein and likely is a consequence of the high level overexpression induced by the rAAV. We also observed nuclear labeling for the GFP protein.

**Figure 2:**
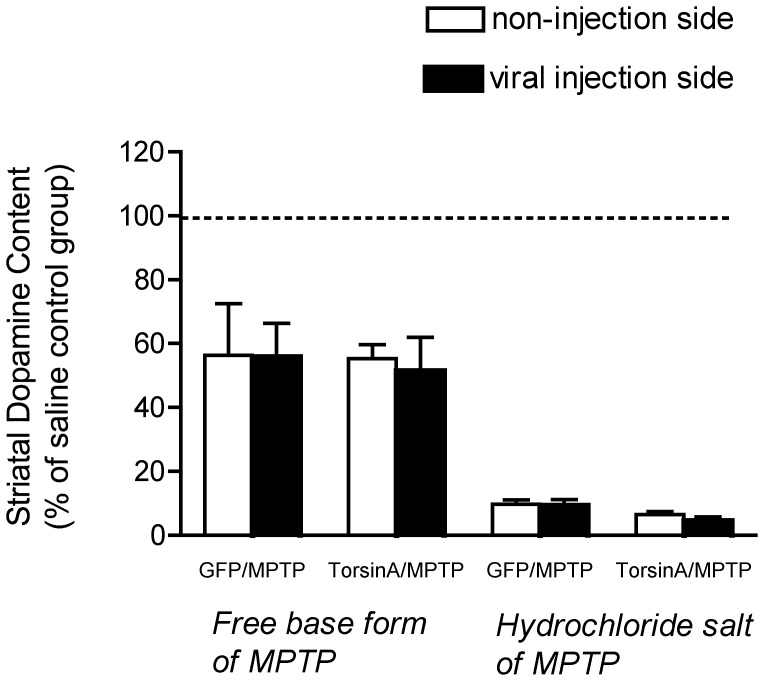
Quantification of striatal DA levels in animals treated with rAAV8-torsinA or rAAV-GFP prior to administration of MPTP. DA levels were determined by HPLC at 14 days after MPTP treatment. The vectors were injected unilaterally, and the left and right striata were analyzed separately to allow comparison of the side injected with rAAV to the contralateral control side in the same animals. All data are normalized to the striatal dopamine content level of saline control group. This experiment was performed twice in two separate groups of animals (n = 10 in each treatment group). The first was conducted using the free base form of MPTP, and produced only a modest degree of DA loss. The second experiment was performed using the hydrochloride salt of MPTP, which produced a more extensive lesion. Data are reported as the mean±SEM. Two-way ANOVA was used to compare differences between virus injection side and non-injection side within each animal group receiving the free base form of MPTP or the hydrochloride salt of MPTP. No significant difference was found in any of the comparisons (P>0.05).

To determine the effect of the MPTP treatment on the striatal content of DA, the frozen striata were analyzed using HPLC. Although our original research plan was only a single replicate of this experiment, we conducted it twice with two separate groups of 20 animals. The reason for this was that in the first replicate, the loss of DA in the striatum was less than expected; this was eventually traced to the use of a form of MPTP (the free base, 1-methyl 4-phenyl 1,2,3,6-tetrahydropyridine, 038K1908, Sigma) that is not well absorbed; this led to a less degree of lesion, and higher mortality from systemic toxicity. The second experiment was conducted with the HCl salt of MPTP (1-methyl 4-phenyl 1,2,3,6-tetrahydropyridine hydrochloride, 038K1908, Sigma) and produced the degree of DA loss expected. Since both experiments are informative, both sets of data on striatal neurochemistry are presented here. For analysis, dopamine content was normalized to the mean of the saline vehicle treated group, and two way ANOVA was used for statistical comparisons. (**Figure 2**). In the free base form of MPTP experiment, the mean loss in striatal DA was about 40%; in the HCl salt form of MPTP experiment, the mean loss in DA content was more than 90%. Neither experiment revealed any evidence for neuroprotection with overexpression of torsinA; indeed, in the free base form of MPTP experiment the data suggest a trend towards worsening of the loss of DA content in the torsinA overexpressing SN (although this difference was not statistically significant).

**Figure 3:**
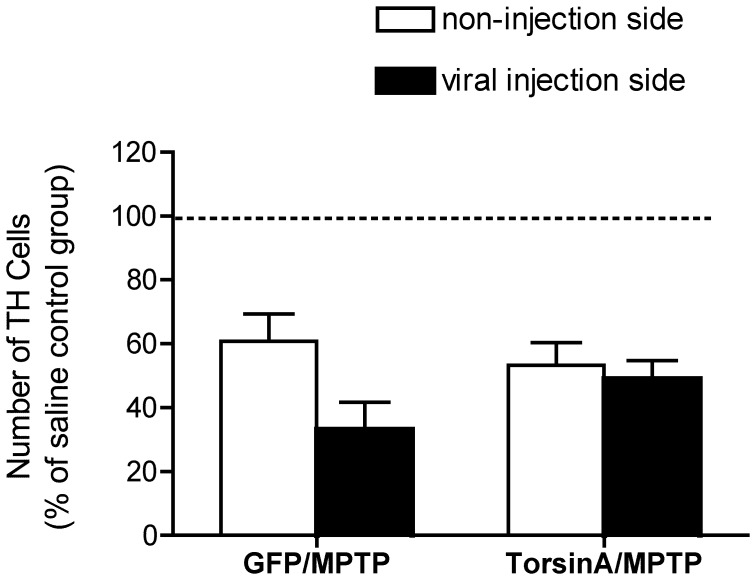
Quantification of TH positive neurons in animals treated with rAAV8-torsinA or rAAV-GFP prior to administration of MPTP. Using sections of the midbrains stained for TH, total numbers of TH positive cells in SN were estimated by unbiased stereology. Neuron numbers in the injection side and the non-injection side were determined separately. Data are normalized to the mean TH positive neuron number of saline control group. A two-way analysis of variance (ANOVA) was used to compare potential differences in the number of TH neurons between left (non-injection side) and right (injection side) sides of each animal groups and differences between the different treatment groups. However, there were no significant differences (P>0.05). The HCl salt form of MPTP was used in this experiment. Group sizes: saline only (n = 10); GFP/MPTP (n = 7); TorsinA/MPTP (n = 6). Data are illustrated as mean±SEM.

We also studied the effect of torsinA overexpression and MPTP treatment on the number of striatal TH positive neurons using an unbiased stereological method (**Figure 3**). A MicroBrightfield instrument was used to count the TH neurons using an optical dissector. We counted TH immunoreactive neurons in the SN of mice transduced with either rAAV8-GFP or rAAV8-torsinA and treated subsequently with MPTP. The animals studied were those from the HCl salt form of MPTP experiment, which had robust loss of DA content, described above. Neuron numbers were normalized to the mean of the saline-treated control group. We found that MPTP led to a loss of TH neurons which was similar in magnitude in both the rAAV-injected and the contralateral uninjected sides and in both rAAV8-torsinA and rAAV8-GFP mouse groups, and there were no statistically significant differences in the TH positive cell numbers among the different treatment groups (P>0.05).

### Effect of Selective Deletion of the Dyt1 Gene from Dopamine Neurons in Vulnerability to MPTP

**Figure 4:**
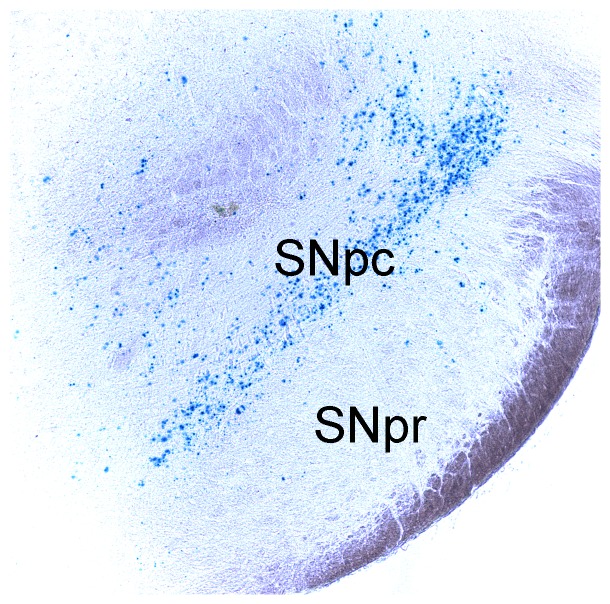
rAAV delivery of Cre recombinase to SN in ROSA reporter mouse. Tissue was processed for LacZ staining, which is induced after successful Cre-mediated genomic recombination in these animals. Blue staining indicates Cre-dependent recombination is present in the majority of SNpc neurons, while no labeling is seen in the adjacent substantia nigra pars reticulata (SNpr).

**Figure 5:**
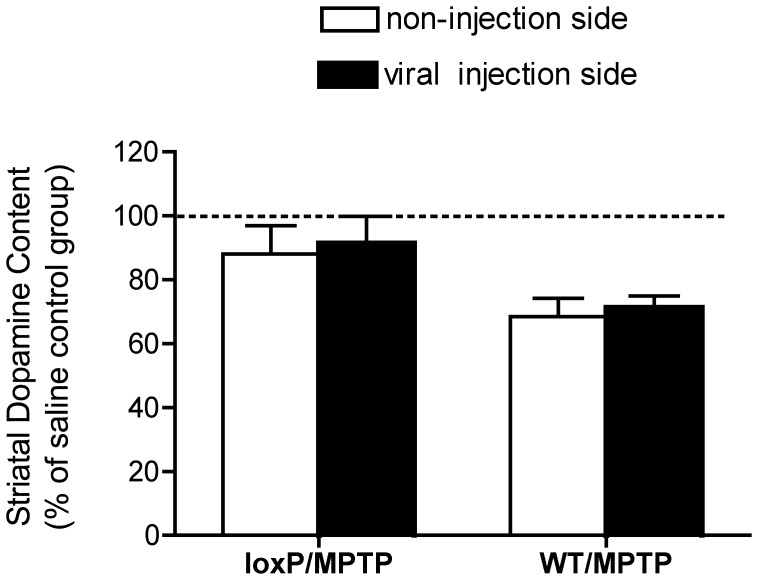
Effect of Cre-mediated deletion of the Dyt1 gene on vulnerability of dopaminergic neurons to MPTP. rAAV-Cre was administered unilaterally to Dyt1-loxP mice and C57/B6 control animals. One month after rAAV treatment, the animals were treated with MPTP. Striatal DA levels were estimated by HPLC at 14 days after MPTP injection. Data are normalized to the striatal DA content of saline-only treatment WT group. Data were reported as the mean±SEM. Two way ANOVA was used to compare potential differences between Cre virus injection side and non-injection side of loxp or WT mice receiving MPTP. However, there was no evidence for enhanced vulnerability after rAAV-Cre in Dyt1-loxP animals. The free base form of MPTP was used in this experiment. Group sizes: WT/Saline (n = 9); loxp/MPTP (n = 10); WT/MPTP (n = 7).

To evaluate the role of endogenous mouse torsinA in protection against MPTP, we performed selective deletion of *Dyt1* (the mouse homolog of the human TOR1A gene) from nigral neurons. Global knockout of *Dyt1* in mice is lethal; here we used a mouse in which the *Dyt1* locus has been flanked with loxP sites (“loxP mice”) [18], and an rAAV2 expressing Cre recombinase to accomplish selective deletion of the Dyt1 gene [19]. We validated this approach using AAV-Cre injection into a ROSA reporter mouse line (**Figure 4**), which demonstrated that the AAV-Cre produced efficient gene deletion within neurons in the SN. The rAAV-Cre was then injected unilaterally into loxP mice. Control animals were WT mice, and were injected with the same active rAAV2-Cre virus. One month after virus injection, the mice were treated with MPTP. In this group of animals, we hypothesized that the knockout of torsinA by rAAV2-Cre would worsen the phenotype; consequently, we used the free base form of MPTP as in the experiments described above. We observed that there was a modest loss of DA content in the WT mice which did not differ between the rAAV injected side and the contralateral non-injected side of the animals. The Dyt1-loxP mice were similar, in that there was no difference in the dopamine content between the rAAV injected side and the contralateral hemisphere; thus, there was no evidence for enhanced dopamine loss after rAAV-Cre injection in the loxP animals. There was a trend towards higher dopamine content in both sides of the loxP animals, but this difference was not statistically significant. (**Figure 5**).

### Effect of Transgenic Overexpression of Human TorsinA on Vulnerability to MPTP

**Figure 6:**
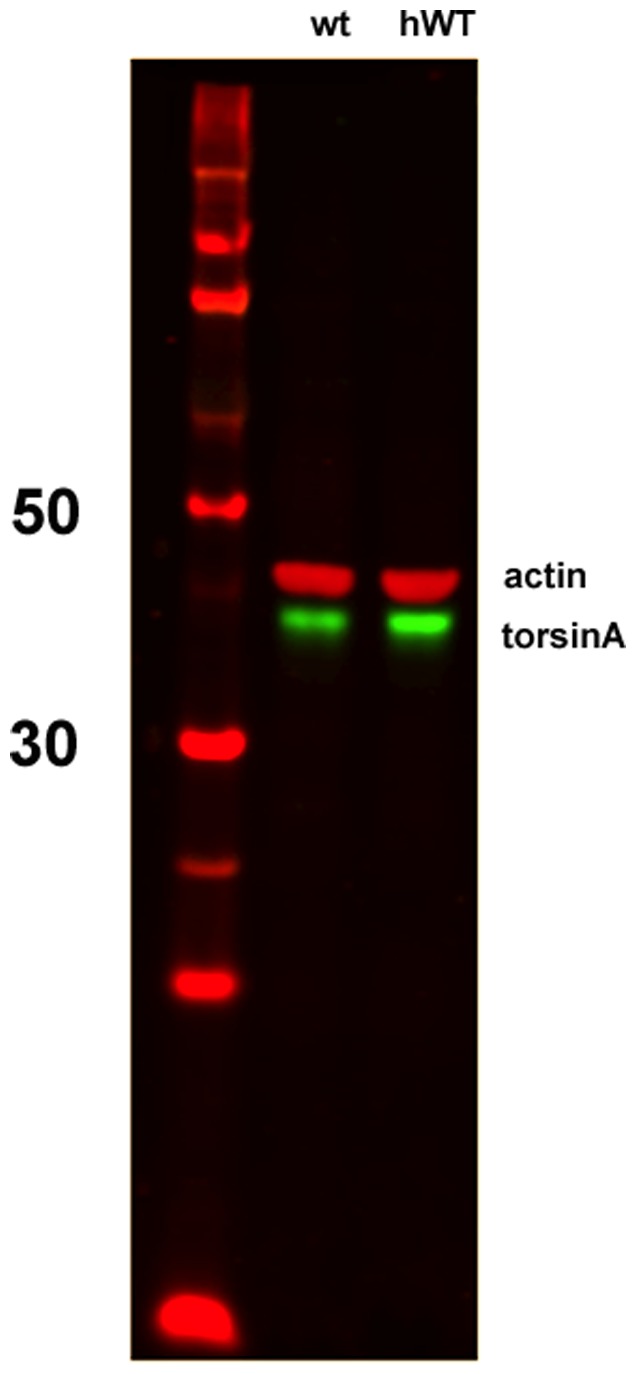
Western Blot Analysis. Blot illustrates abundance of torsinA (lower band, in green) in hWT transgenic mice (right lane) and littermate controls (center lane). Left lane contains size markers, with 50 and 30 kd positions labeled. Upper bands (red) are actin controls run simultaneously. hWT mice show a greater intensity of torsinA; when intensities are measured quantitatively and normalized to actin, the ratio of hWT to control is approximately 1.5.

We used an existing line of torsinA transgenic mice hWT, to determine whether this approach to torsinA overexpression conferred neuroprotection against MPTP. In contrast to the rAAV overexpression approach described above, these transgenic animals have widespread expression of torsinA (under control of a viral CMV promoter), and express the protein from an early age. In addition, they express the human form of torsinA, in addition to the endogenous mouse protein which is also present. We [20] and others [21] have previously examined the extent of torsinA expression in these animals. Sharma et al. [20] reported that these animals expressed torsinA at a level of about 2.3 fold that of control mice. Zhao et al. [21] evaluated mRNA expression, and found that human torsinA transcript could be detected in these mice, although it was at a lower level than was found in human brain. Because genetic drift is possible, we re-evaluated torsinA expression in the striatum using animals from our current colony, and observed a level of torsinA in these animals that was approximately 1.5 that of controls (Figure 6).

**Figure 7:**
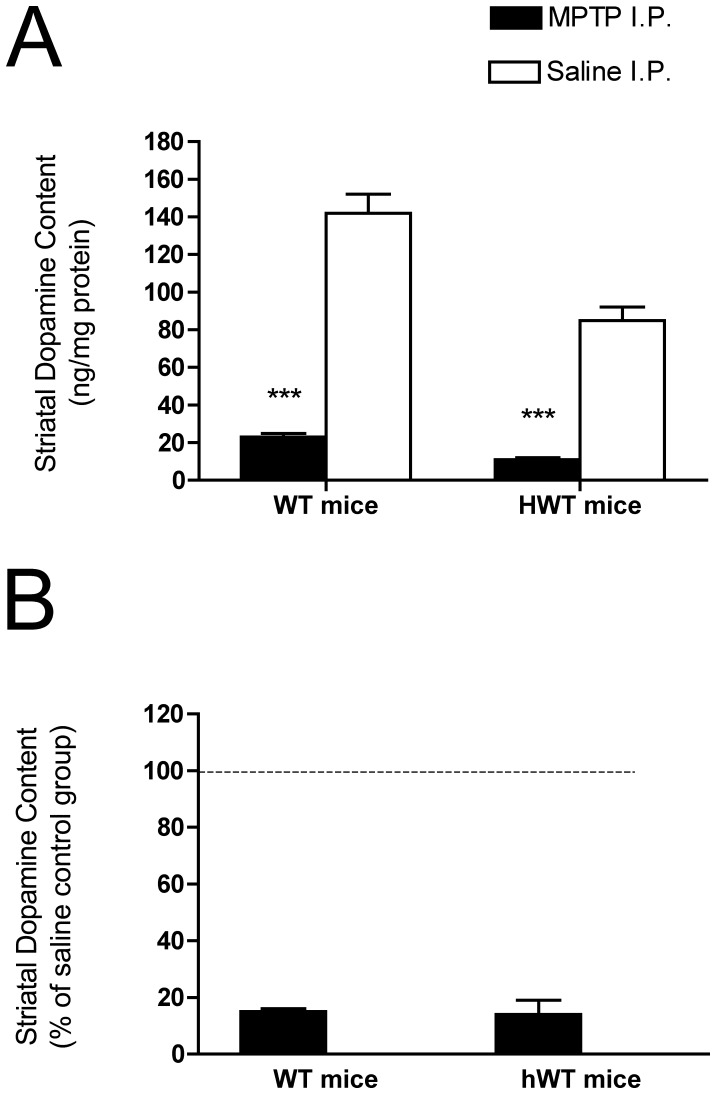
Quantification of striatal DA levels after MPTP or saline injection of WT, and hWT mice. Striatal DA levels were estimated by HPLC in these mice at 14 days after MPTP or saline injection. Data were reported as the mean±SEM. Two-way ANOVA (Figure 7A) and one-way ANOVA (Figure 7B) were used to assess significance. In Figure 7B, the differences in the striatal DA level between the saline and MPTP treatment groups are expressed as a percentage of the matched saline control groups. There was a significant reduction in striatal DA level in each of the MPTP treatment groups compared with saline injection groups (***P<0.001) but the effect did not differ by genotype. The HCl salt form of MPTP was used in this experiment. Group sizes: WT/MPTP (n = 9); WT/Saline (n = 10); HWT/MPTP (n = 9) HWT/Saline (n = 10).

Groups of hWT transgenic mice along with control wild type mice (non-transgenic littermates) were received MPTP or saline vehicle I.P. injection. At 14 days post-treatment of MPTP or saline, we measured the striatal DA content. The level of DA in the striatum of the MPTP treatment groups was markedly lower than that of the saline treatment groups (**Figure 7A**). While there were baseline differences in the absolute content of DA in the different mouse lines, when expressed as a percentage of the saline control groups the MPTP-induced DA loss was similar in both groups (**Figure 7B**). Because of the uniformity of the DA loss and the severity of the lesions in, unbiased stereology was not performed in this set of experiments.

### Effect of Transgenic Overexpression of Human TorsinA on Vulnerability to rAAV-Induced α-SYN Overexpression

All of the preceding experiments were performed using acute intoxication with MPTP. This toxin is often used as a model of PD; it is linked mechanistically to the disease because it is an inhibitor of complex I of the mitochondrial respiratory chain, and accidental ingestion of MPTP can cause human parkinsonism [22]. In recent years, however, the relevance of MPTP to sporadic PD has been questioned. We also studied an alternative model in which overexpression of alpha-synuclein, produced using an rAAV vector, leads to progressive dopaminergic degeneration [17].

**Figure 8:**
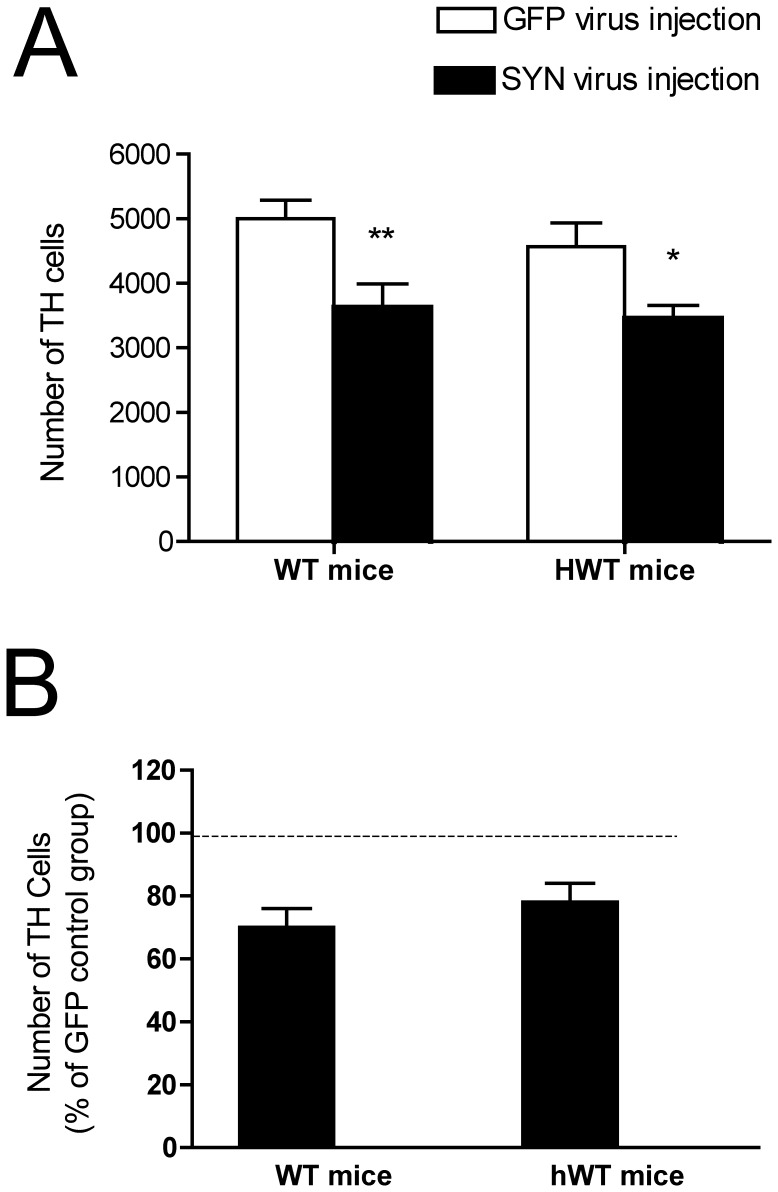
Quantification of TH positive cells in SN of rAAV-SYN and rAAV-GFP injected WT and hWT mice. Total numbers of TH positive cells at 6 months after rAAV-SYN and rAAV-GFP injection were estimated by unbiased stereology. Data were reported as the mean±SEM. Figure 8B shows the differences in the number of TH cells between the rAAV-SYN and rAAV-GFP treatment groups expressed as a percentage of the AAV-GFP control groups. Two-way ANOVA (Figure 8A) and one-way ANOVA (Figure 8B) were used to assess significance. Bars with one or more stars are statistically significantly different from each other (*P<0.05; **P<0.01). Group N’s: WT-GFP (n = 10); WT-SYN (n = 9); HWT-GFP (n = 9); HWT-SYN (n = 8).

A total of 20 male hWT and 20 control (non-transgenic littermate) mice were injected unilaterally with either rAAV2-SYN or rAAV2-GFP. At 6 months post-treatment of rAAV2-SYN or rAAV2-GFP virus injection, we measured the TH positive neuron number by unbiased stereology. The number of the TH positive cell in SN of the rAAV2-SYN injected control (WT) animals was reduced by about 25%, similar to previously published experiments using this method. A similar degree of loss was observed in the hWT group, and no difference between the genotypes could be detected. (**Figure 8**).

## Discussion

In this study we evaluated the effects of the protein torsinA in several mouse models of PD, based either on intoxication with MPTP or overexpression of alpha-synuclein induced by an rAAV. We found no evidence for a protective effect of torsinA in any of these experiments. Using the acute MPTP model, neither rAAV induced nor transgenic overexpression of torsinA was protective, while deletion of the *Dyt1* gene from dopamine neurons using AAV-Cre in a Dyt1-loxP mouse did not enhance sensitivity to MPTP. Transgenic expression of human torsinA also failed to protect against loss of TH positive neurons induced by rAAV mediated overexpression of alpha-synuclein. These negative data raise two critical questions about these experiments: have we effectively modified the expression of torsinA, and are the models themselves predictive of the potential effects of such manipulations in human PD?

We used several different approaches to modify torsinA expression in these mouse models. In the first set of experiments, we used an rAAV viral vector to induce high level expression of the protein within neurons in the substantia nigra. The immunohistochemical data indicate that this vector did achieve the goal of enhancing neuronal expression of torsinA in neurons in the substantia nigra (**Figure 1**). Indeed, the antibody used in these experiments is specific for torsinA, but has relatively low sensitivity when used for immunohistochemistry; thus, we infer that robust staining indicated that the degree of protein expression induced by the rAAV-torsinA must be several times higher than the level of the endogenous mouse protein. This high level expression likely accounts for the presence of torsinA staining in the nucleus of transfected neurons, as the endogenous protein is exclusively cytoplasmic. The expression of torsinA in the transgenic model (hWT) has been reported previously [20], [21]. Because of the possibility of genetic drift in colonies of mice, we reassessed the abundance of torsinA in these animals and confirmed that it remains elevated, although the abundance we observed was not a large as previously described, with about a 50% increase in the transgenic animals compared to control. Thus, this approach induces a much more modest enhancement of torsinA than the rAAV strategy. The degree of knockdown achieved using the AAV-Cre in the LoxP animals is more difficult to assess directly, because of the difficulty in obtaining reliable staining of endogenous torsinA in non-transgenic mice. Nevertheless, the data from the ROSA reporter mouse suggest that this AAV-Cre induces very complete recombination of the target loxP sites.

With regard to the specific mouse models of PD employed, there is at present no consensus within the field as to a single ‘optimal’ model to study potential neuroprotective agents, and most have suggested studying a range of models based on different pathophysiological mechanisms [2]. The acute MPTP paradigm we employed in these studies has been widely used in prior work [22]. The neurotoxicity of MPTP was discovered after accidental human exposure led to irreversible parkinsonism, and the toxin does reproduce many features of the disease, including the relative selectivity for dopaminergic neurons, neuro-inflammatory responses, and inhibition of Complex I of the mitochondrial chain. On the other hand, there is no convincing evidence for a significant role of MPTP in human PD, and the rapid destruction of dopamine neurons induced by acute administration is quite different from the gradual degeneration seen in human PD. There are several alternative models based on more chronic administration of lower doses of MPTP, and these may be more faithful models of the human condition. It is possible that effects of torsinA might be detected by the use of one of these alternative models.

In addition to MPTP, we have also used an rAAV-SYN model of PD. The relationship of α-syn overexpression to human PD is well established; both mutations in α-syn as well as gene duplication of the α-syn locus leading to overexpression are established genetic causes of human PD, and aggregates of α-syn are a universal feature of the disease [23]. A number of transgenic models overexpressing α-syn have been described, but for the most part these models either lack evidence of neurodegeneration, or have patterns of degeneration which are quite different from human PD [24]. The rAAV vector approach induces high-level α-syn expression in adult animals, and induces progressive dopamine neuron degeneration in mice, rats, and non-human primates [25]. The extent of degeneration observed in mice is somewhat less than in these other species; here we observed loss of about 25% of the TH positive nigral neurons at 6 months after injection, similar to that reported in previous studies [17].

The goal of this project was to seek evidence that would support or refute the utility of torsinA as a target of PD therapy. It is important to appreciate that target validation is not a linear process, but rather a probabilistic exercise. This is especially true in PD, because there is at present no single cellular or animal model with established value in predicting the outcome of a human neuroprotective treatment. We have studied models based on two distinct mechanisms, MPTP intoxication and α-syn overexpression, and used different approaches to modify torsinA. We cannot, of course, exclude the possibility that study of other types of models or strategies might reveal a neuroprotective signal with torsinA. Nevertheless, our failure to identify protective effects of torsinA in these several mouse models examined does seem to diminish the likelihood that this molecule can be successfully translated into a human neuroprotective target in PD. This result is of course useful, because it suggests that efforts and resources might better be devoted to other potential targets for which there is substantial evidence for neuroprotection *in vivo* across a range of model systems [1].

## Methods

### Animals and Treatments

All animal procedures were approved by the Institutional Animal Care and Use Committee of the University of Alabama at Birmingham and conducted in accordance with National Institutes of Health guidelines. Mice were housed under 12 hour light/12 hour dark cycle and were provided standard mouse diet. C57BL/6 wild type mice were used as control animals in these experiments, and all of the transgenic and genetic modified lines have been extensively backcrossed into this background. All of the animals used in this study were male and aged between 8–12 weeks. Groups sizes for each experiment were generally at least 10 animals per treatment condition; because of peri-operative and MPTP-related mortality, the number actually available for analysis was in some cases less than 10. The group size for each of the data sets is included in the figure legends.

Two lines of genetically modified mice were used in these studies. We used an existing transgenic mouse line, hWT, which expresses the wild type form of human torsinA protein under a CMV promoter. These mice have been extensively characterized previously, both by our lab [20] as well as others [21]. These animals have no obvious motor abnormality or neurodegeneration [26]. To perform selective deletion of Dyt1 from nigral neurons, we used an existing Dyt1-loxP mouse line [18] (provided by Dr. Yuqing Li, University of Florida).

### Western Blotting

Brain tissue was removed from control and transgenic mice and processed for Western blotting. In brief, samples were homogenized in lysis buffer (50 mM Tris, 250 mM sodium chloride, 2 mM sodium orthovanidate, 5 mM EDTA, 1 mM sodium fluoride, 0.1% deoxycholate, 0.02% sodium azide, 1% Triton X-100, 1% SDS) with protease inhibitors and then spun at 14,000×*g* for 10 min at 4°C. Sample protein concentration was then determined using a BCA protein assay. Laemmli sample buffer (2.5% SDS, 50 mM Tris, 0.015% bromophenol blue, 10% glycerol, 2% B-mercaptoethanol) was added and samples were boiled for approximately 10 min. Fifty ug of protein was run on a 4–12% gradient Bis-Tris gel (Invitrogen) and transferred to nitrocellulose using semi-dry BioRad transblotters (Hercules, CA). Blots were exposed to LiCor blocking buffer (Lincoln, NE) for 1 h at room temperature, then probed with torsinA-specific polyclonal antibody (1∶1000, Abcam) and B-actin-specific monoclonal antibody (1∶10000, Sigma, St. Louis, MO) in the same buffer. Appropriate LiCor fluorescent secondary antibodies (IR-Dye 670 or 880cw, Lincoln, NE) were then applied for 1 h at room temperature. Membranes were imaged using a LiCor Odyssey scanner. Boxes were manually drawn around each band of interest and raw intensity was measured using Odyssey 3.0 analytical software (LiCor, Lincoln, NE). Values for TorsinA were divided by B-actin values in the same sample to derive an adjusted measure of protein expression.

### rAAV Viral Vectors

Five different kinds of rAAV viral vector of sterotype 2 or 8 were used in this study. To produced gene deletion in the Dyt1-loxP mice, we used an rAAV2-Cre which has been previously described [19] (a kind gift from Dr. Tom Scammell of Harvard University) which was repackaged in our laboratory with a viral titer of 3.0×10^9^ viral genome/ml. The rAAV2-SYN vector used was originally developed by Dr. Pamela McLean of Harvard University, and has been described previously [17]. It contains expression cassettes for both human α-syn and GFP, separated by an internal ribosomal entry site (IRES). This virus was repackaged in our lab at viral titer 6.0×10^10^ viral genome/ml. As a control for experiments with this vector we used a similar rAAV2 containing only the GFP cassette (1.44×10^11^ viral genome/ml). An rAAV8-torsinA expressing normal human torsinA with a viral titer of 3.77×10^12^ viral genome/ml, along with a control rAAV8-GFP (2.6×10^12^ viral genome/ml) were provided by Drs. Miguel Sena and Xandra Breakefield of Massachusetts General Hospital (MGH).

Viral vector injections were performed under isoflurane anesthesia using stereotaxic coordinates: antero-posterior, −3.2 mm from bregma, medio-lateral, −1.2 mm from midline and dorso-ventral, −4.6 mm from the dura. Mice received a volume of 2 µl viral vector at a flow rate of 0.25 µl/minute over eight minutes, followed by four minutes for diffusion before the syringe was slowly removed.

### MPTP Treatment

The animals were received I.P. injection of MPTP at every 2 hours for a total of four doses over an 8 hour period in 1 day (17 mg/kg in 0.9% saline per dose×4), under isoflurane anesthesia. In the control groups the animals were received I.P. injection of 0.9% saline at every 2 hours for four times over an 8 hour period in 1 day, under isoflurane anesthesia. Two forms of MPTP were used in this study. The free base form of MPTP (1-methyl 4-phenyl 1,2,3,6-tetrahydropyridine, 038K1908, Sigma) and the HCl salt form of MPTP (1-methyl 4-phenyl 1,2,3,6-tetrahydropyridine hydrochloride, 038K1908, Sigma). The MPTP forms used for each of the data sets is included in the figure legends.

### Tissue Preparation

For MPTP studies, animals were euthanized at 2 weeks following MPTP treatment by decapitation under deep anesthesia. The brain was removed and separated into forebrain and midbrain. The forebrains were dissected to isolate the striata, which were rapidly frozen on dry ice. These samples were shipped on dry ice to the Neurochemistry Core Lab., Vanderbilt University Medical Center, Nashville, TN where the content of DA and metabolites were measured by HPLC. The midbrains were fixed by immersion in 4% paraformaldehyde for 48 hours before cryopreservation by suspension in 30% sucrose in 0.1 M phosphate buffered saline (PBS) for 24 hours and then stored frozen.

For studies employing the AAV-SYN vector, animals were euthanized at 24 weeks following rAAV2-SYN or rAAV2-GFP injection by transcardiac perfusion with 4% paraformaldehyde in PBS under deep anesthesia. Brains were removed and post-fixed in the same fixative for two hours at room temperature followed by immersion in 30% sucrose in PBS for 24 hours. The brains were then stored frozen.

Fixed brains from both types of experiments were sectioned coronally at a thickness of 40 µm using a sliding microtome and collected as free-floating sections. They were stored in 50% glycerol in PBS at −20°C.

### Bright-field Immunohistochemistry

For determination of the number of TH positive neurons, every sixth section from the midbrain of the animals was stained using 3,3′-diaminobenzidine (DAB) as a chromagen. Briefly, the floating sections were treated with 0.3% hydrogen peroxide and blocked in PBS containing 3% normal goat serum. The sections were incubated with a primary antibody, rabbit anti- tyrosine hydroxylase (TH) (Pelfreeze Biologicals, Rogers, AR) at 1∶1000 dilution in the blocking solution at 4°C overnight. Sections were then incubated with appropriate secondary antibody, biotinylated goat anti-rabbit (Vector Labs, Burlingame, CA) diluted at 1∶400 for 1 hour at room temperature followed by amplification with the ABC system (PK-7100, Vector Labs) for 30 minutes and then followed by peroxidase with the DAB substrate kit (SK-4100, Vector Labs) for 4–6 minutes. Immunolabeled sections were mounted on slides (SuperFrost Plus; Fisher Scientific, Pittsburgh, PA) and dehydrated with xylene and then coverslipped with DPX mountant (Fluka).

### Double Fluorescence Immunocytochemistry

Free-floating SN sections were double stained with TH antibody diluted 1∶1,000 (Pelfreez, P401-010L0) and mouse anti-human torsinA-DM2A8 antibody diluted 1∶200 (kindly provided by Xandra Breakefield, MGH), mouse anti-Cre antibody diluted 1∶400 (Covance 106p) or GFP antibody diluted 1∶2000 (Abcam, Cambridge, MA) overnight at 4°C. Tissue was then washed and appropriate Alexa-488-conjugated secondary antibodies diluted 1∶500 (Jackson Immunoresearch, West Grove, PA) and CY3-conjugated secondary antibodies diluted 1∶500 (Jackson Immunoresearch, West Grove, PA) were used at room temperature for 2 hours. All sections were then washed, mounted onto Superfrost slides (Fisher Scientific, Pittsburgh, PA, USA), and coverslipped with Vectashield aqueous mounting medium (Vector Laboratories, Burlingame, CA, USA).

### Stereological Quantitation of DA Neurons

TH stained cells were counted by the optical fractionator method using Stereoinvestigator 7.0 software from MBF Biosciences (Microbrightfield, Inc, Williston, VT). The operator was blinded to the group identification. Briefly, coded slides were scanned on the stage of a modified Olympus BX51 bright-field microscope using a low-power objective, and SN on the injected and non-injected sides were contoured. Every sixth section spanning the entire SN was included in the counting procedure. Sections covering the rostro-caudal extent of the SN (usually 5 sections per animal) were counted and the number weighted section thickness was used to correct for variations in tissue thickness at different sites.

### LacZ Staining

ROSA reporter mice were deeply anesthetized with pentobarbital and transcardially perfused with 4% paraformaldehyde in 0.1 M PBS. Brains were removed and post-fixed in the same fixative at 4C for 6 hrs, and then soaked in 30% sucrose in PBS for 72 hrs at 4C. The brains were frozen and sectioned coronally at a thickness of 50 µm using a sliding microtome. Sections were washed for 10 min×3 in 0.1 M phosphate buffer and 10 min×3 in 0.1 M phosphate buffer, 2 mM MgCl2, 0.01% sodium deoxycholate, and 0.02% NP-40. Sections were stained with x-gal solution overnight at 37°C before being mounted and coverslipped with Kaiser’s glycerol jelly. Slides were viewed using a Nikon Eclipse microscope (E800 M).

### Confocal Images

Confocal images were captured using a Leica TCS-SP5 laser scanning confocal microscope. The images were processed using the Leica software and exported as TIFF files and processed using Adobe Photoshop CS2.

### Statistical Analyses

All data are expressed as mean±SEM. Stereology and striatal DA content data were analyzed by using a one way or two-way analysis of variance (ANOVA) with post hoc tests (Bonferroni method). “ Significance was assigned at the p<0.05 level. Prism 4 software was used to conduct these analyses (Graphpad Inc., San Diego, CA, USA).
